# Effects of cobalt-histidine absorbent on aerobic denitrification by *Paracoccus versutus* LYM

**DOI:** 10.1186/s13568-019-0927-x

**Published:** 2019-12-17

**Authors:** Chaoyue Sun, Yu Zhang, Zhenping Qu, Jiti Zhou

**Affiliations:** Key Laboratory of Industrial Ecology and Environmental Engineering (Ministry of Education), School of Environmental Science and Technology, Dalian University of Technology, Dalian, 116024 China

**Keywords:** Cobalt-histidine absorbent, Inhibition, Aerobic denitrification, *Paracoccus versutus*

## Abstract

To overcome the problem that ferrous complexes are easily oxidized by O_2_ and then lose NO binding ability in the chemical absorption-biological reduction (CABR) process, cobalt(II)-histidine [Co(II)His] was proposed as an alternative. To evaluate the applicability of Co(II)His, the effects of CoHis absorbent on the aerobic denitrification by *Paracoccus versutus* LYM were investigated. Results indicated that His significantly promoted nitrite reduction. The inhibition effects of CoHis absorbent could be substantially alleviated by increasing the initial His/Co^2+^ to 4 or higher. CoHis with concentrations of 4, 8, 12, 16 and 20 mM presented no distinct effect on nitrite reduction, but slightly inhibited the reduction of nitrate, resulting in longer lag of nitrate reduction, and obviously promoted the growth of strain LYM. In the presence of 5, 10, 15 and 20 mM CoHis absorbent, the main denitrification product was N_2_ (not less than 95.0%). This study is of significance in verifying the applicability of Co(II)His in the CABR process, and provides a referable CoHis absorbent concentration as 20 mM with an initial His/Co^2+^ of 4 for the future experiments.

## Introduction

As a cost-effective and eco-friendly process for nitric oxide (NO) removal, the chemical absorption-biological reduction (CABR) flue gas denitrification technology is based on wet absorption of NO using ferrous complexes, especially ferrous ethylenediaminetetraacetate [Fe(II)EDTA], to improve the NO absorption into scrubbing liquid, and biological reduction regeneration of chelate agent to reduce operation costs (Zhang et al. 2014, 2018; Zhao et al. 2016). However, typical flue gas after the traditional flue gas desulfurization process is estimated to contain 3–8% (v/v) O_2_ (Zhang et al. 2008). The ferrous complexes are easily oxidized by the O_2_ and form ferric complexes (Seibig and van Eldik 1997), which are not capable of binding NO (Zhang et al. 2014). Besides, traditional biological denitrification process is under anoxic condition. The denitrifying activity during biological reduction process in the CABR technology would be largely suppressed due to the sensitivity of denitrifying reductase to the presence of O_2_.

According to recent researches, it is indicated that cobalt(II) complexes might be promising alternatives to ferrous complexes in the CABR technology, and the benefits of cobalt(II) complexes mainly come from two aspects. Firstly, some cobalt complexes, especially hexamminecobalt(II) [Co(NH_3_)_6_^2+^] (Long et al. 2017; Yu and Tan 2014), can absorb NO and O_2_ simultaneously to form nitrosyl complexes (reaction 1) and binuclear cobalt complex ions with bridging dioxygen (reaction 2).This property makes the oxidation of cobalt(II) complexes to be considerably slower than ferrous complexes. Secondly, the O_2_ existing in the gas phase aids the abatement of NO greatly (Long et al. 2017). This is because the binuclear cobalt complexes will further react with the nitrosyl products to form nitro complexes (reaction 3), which will eventually be transformed into soluble nitrate and nitrite (reaction 4). The flue gas denitrification process of Co(NH_3_)_6_^2+^ is given below as an example.

1 $${\text{Co}}\left( {{\text{NH}}_{ 3} } \right)_{ 6}^{ 2+ } + {\text{NO}} \to \left[ {{\text{Co}}\left( {{\text{NH}}_{ 3} } \right)_{ 5} {\text{NO}}} \right]^{ 2+ } + {\text{NH}}_{ 3}$$

2 $$2 {\text{Co}}\left( {{\text{NH}}_{ 3} } \right)_{ 6}^{ 2+ } + {\text{O}}_{ 2} \to \left[ {\left( {{\text{NH}}_{ 3} } \right)_{ 5} {\text{Co}}{-}{\text{O}}{-}{\text{O}}{-}{\text{Co}}\left( {{\text{NH}}_{ 3} } \right)_{ 5} } \right]^{ 4+ }$$

3 $$\left[ {\left( {{\text{NH}}_{ 3} } \right)_{ 5} {\text{Co}}{-}{\text{O}}{-}{\text{O}}{-}{\text{Co}}\left( {{\text{NH}}_{ 3} } \right)_{ 5} } \right]^{ 4+ } + {\text{H}}_{ 2} {\text{O}} + 2 {\text{NH}}_{ 3} + \left[ {{\text{Co}}\left( {{\text{NH}}_{ 3} } \right)_{ 5} {\text{NO}}} \right]^{ 2+ } \to 2 {\text{Co}}\left( {{\text{NH}}_{ 3} } \right)_{ 6}^{ 3+ } + 2 {\text{OH}}^{ - } + \left[ {{\text{Co}}\left( {{\text{NH}}_{ 3} } \right)_{ 5} {\text{NO}}_{ 2} } \right]^{ 2+ }$$

4 $$2\left[ {{\text{Co}}\left( {{\text{NH}}_{ 3} } \right)_{ 5} {\text{NO}}_{ 2} } \right]^{ 2+ } + {\text{H}}_{ 2} {\text{O}} + 2 {\text{NH}}_{ 3} \to {\text{NH}}_{ 4} {\text{NO}}_{ 2} + {\text{NH}}_{ 4} {\text{NO}}_{ 3} + 2 {\text{Co}}\left( {{\text{NH}}_{ 3} } \right)_{ 6}^{ 2+ }$$

These merits of O_2_ adverse impact resistance provide theoretical advantages of cobalt(II) complexes over ferrous complexes in the CABR process. While to the best of our knowledge, such kinds of experiments have been lacking. And several issues must be addressed before cobalt complexes could be granted as applicable absorbent in the standard CABR process. Firstly, a suitable cobalt complex must be found. The most renowned cobalt(II) complex is Co(NH_3_)_6_^2+^, but its high pH functional environment (Yu and Tan 2014) is not suitable for the CABR process. Fortunately, our previous study (Sun and Zhang 2018) finds that cobalt(II)-histidine [Co(II)His] can absorb both NO and O_2_ in the neutral condition, which is required in the CABR process.

In the CABR processes, to reduce operation costs and prevent secondary pollution, the produced Fe(II)EDTA-NO and Fe(III)EDTA during NO absorption process were reduced to Fe(II)EDTA by biological reduction process to regenerate the absorbent. In the flue gas denitrification process of Co(II)His, as can be inferred from reactions 1–4, nitrate and nitrite might be the final product. Additionally, though Co(II)His have an excellent reversible oxygenation ability, it is still oxidized slowly by autoxidation effect and form cobalt(III)-histidine [Co(III)His], thus losing absorption capability of NO and O_2_ (Zhang et al. 2016). Hence, the biological reduction of nitrate, nitrite, and Co(III)His under aerobic condition would be another important issue to be studied. As cobalt is a heavy metal, the impact and toxicity of the cobalt complex absorbent on biological reduction of nitrate and nitrite under aerobic atmosphere should be studied and disposed, and this is the main theme of this research.

The importance of metal speciation in the toxicity of heavy metal has been confirmed by many researches. Hu et al. (2002), Semerci and Çeçen (2007) prove that the deleterious effects of heavy metals depend on free cation concentration instead of total metal concentration, and the former can be modulated by adding excessive strong chelating agents (e.g., EDTA). Accordingly, the Co(II)His absorbent used in the present study was prepared based on the reversible reaction of Co^2+^ with His (reaction 5) (Burk et al. 1946). In order to reduce the adverse impact of Co(II)His absorbent by suppressing the Co^2+^ concentration, the initial His to Co^2+^ ratio or ratios (His/Co^2+^) should be higher than the stoichiometric ratio of 2 according to the Le Châtelier’s principle. As there was substantial residual free His in the Co(II)His absorbent, the effects of His on the removal of nitrate and nitrite could not be ignored. In addition, Gui et al. (2017) confirms that some heavy metals restrain aerobic denitrifying activity of *Pseudomonas stutzeri* PCN-1 and lead to the accumulation of nitrous oxide (N_2_O), a potent greenhouse gas (Carreira et al. 2017). Thus, the gas product analysis of the aerobic denitrification process under Co(II)His absorbent was also important.

5 $${\text{Co}}^{ 2+ } + 2 {\text{His}}{ \leftrightharpoons }{\text{Co}}\left( {\text{His}} \right)_{ 2} + 2 {\text{H}}^{ + }$$

The *Paracoccus versutus* LYM isolated by our research group with denitrification capability under aerobic environment (Zhang et al. 2015) was used in this research. Besides, CoHis absorbent, i.e., absorbent contained both Co(II)His and Co(III)His, was used instead of Co(II)His absorbent in following description. As a whole, the present study was conducted to determine the effects of (a) His, initial His/Co^2+^ and CoHis absorbent on the removal of nitrate and nitrite by *P. versutus* LYM, (b) CoHis absorbent on gas products of aerobic denitrification by *P. versutus* LYM.

## Materials and methods

### Chemicals, bacterial strain and culture conditions

l-Histidine (His, C_6_H_9_N_3_O_2_, 99%) was purchased from Dalian Meilun Biological Technology Co., Ltd. (Dalian, China). Cobalt chloride (CoCl_2_·6H_2_O, 99.0%) was purchased from Tianjin Guangfu Fine Chemical Research Institute (Tianjin, China). Oxygen (O_2_, 99.99%) was obtained from Dalian Guangming Gas Company (Dalian, China). All other chemicals were of analytical grade, commercially available, and used without further purification.

Strain LYM, identified as *Paracoccus versutus* by 16S rRNA amplification and sequencing, was isolated from seabed sludge. This strain (GenBank accession No.JQ328185) was deposited in Guangdong Culture Collection Center, and the collection number of this strain was GIMCC 1.487.

Strain LYM was routinely cultured in Luria–Bertani (LB) broth medium aerobically at 30 °C in a rotary incubation shaker (150 rpm) until the cell optical density (OD_660_) reached approximately 2.8. Cells were harvested by centrifugation (10,000 rpm, 8 min) and washed twice with sterile phosphate-buffered saline (PBS, 20 mM, pH 7.0). The cell pellets were then used in the following studies.

The basal medium consisted of (unless specified otherwise): MgSO_4_·7H_2_O (0.1 g L^−1^), NH_4_Cl (0.535 g L^−1^), Na_2_HPO_4_·12H_2_O (5.73 g L^−1^), KH_2_PO_4_ (0.54 g L^−1^), and trace elements solution (1 mL L^−1^). The trace elements solution contained (g L^−1^): EDTA (50), ZnSO_4_ (22), CaCl_2_ (5.5), MnCl_2_·4H_2_O (5.06), FeSO_4_·7H_2_O (50), (NH_4_)_6_Mo_7_O_24_·4H_2_O (1.1), CuSO_4_·5H_2_O (1.57) and CoCl_2_·6H_2_O (1.61) (Robertson and Kuenen 1992). Sodium lactate was used as sole carbon source, whose amount depended on the change of external total nitrogen with a carbon to nitrogen mass ratio fixed as 15. The pH for all the media was adjusted to approximately 7.2. The media used were all autoclaved before use (20 min at 121 °C).

### Aerobic denitrification experiments

Aerobic denitrification experiments were conducted in 250 mL conical flasks in a shaking incubator (150 rpm at 30 °C; initial dissolved oxygen 8 mg/L). The total volume of liquid was 100 mL. The initial cell concentrations were (0.28–0.33) g dry cell weight (DCW)/L. To determine the effects of His on aerobic denitrification, assays were conducted with 10 mM nitrate (or nitrite) and varying His concentrations (10, 20, 30, 40 and 60 mM) in the basal medium. Similarly, to assess the effects of initial His/Co^2+^ on aerobic denitrification, assays were conducted with 10 mM nitrate (or nitrite), 5 mM CoCl_2_·6H_2_O and varying His concentrations (10, 15, 20, 25, and 30 mM) in the basal medium. To evaluate the effects of CoHis on aerobic denitrification, 15 mM nitrate (or nitrite) and different concentrations of CoHis absorbent (4, 8, 12, 16 and 20 mM with an initial His/Co^2+^ of 4) were added into the basal medium. Samples were taken periodically for the measurement of nitrate, nitrite, cobalt(II) and cells. Assays with biomass but without His (or CoHis absorbent) served as control group (CG). Assays without biomass and His (or CoHis absorbent) served as sterile control. Experiments and controls were performed in two or three independent replicates. Additionally, to investigate the oxidation process of Fe(II) and cobalt(II) by dissolved oxygen, the oxidation experiments of Fe(II)EDTA (5, 10 and 20 mM) and Co(II)His (5 and 10 mM with an initial His/Co^2+^ of 4) were conducted in 250 mL conical flasks under 150 rpm without biomass. The concentrations of Fe(II) and cobalt(II) were measured periodically.

### Analysis of gas products

The gas products analysis experiments were carried out in 250 mL glass serum bottles in a shaking incubator (150 rpm at 30 °C). Varying concentrations of CoHis (5, 10, 15 and 20 mM with an initial His/Co^2+^ of 4) and 10 mM nitrate were added into the basal medium. The total volume of liquid was 100 mL, and the headspace was replaced with O_2_. The initial cell concentration was 0.30 g DCW/L. The gas samples (250 μL) were periodically sampled by gas tight syringe to detect N_2_O and N_2_ by gas chromatography. Assay with biomass but without CoHis absorbent served as CG. Assay without biomass and CoHis absorbent served as sterile control. Experiments and controls were performed in two independent replicates.

### Analysis methods

OD_660_ was assayed at 660 nm using the spectrophotometer (UV-560, JASCO, Japan). The concentrations of nitrate and nitrite were determined according to the standard methods (Zhang et al. 2015). Cobalt(II) concentration was determined by spectrophotometry (UV-560, JASCO, Japan) according to the method used by Long et al. (2017). Biomass growth was quantified by measuring the bacterial protein concentration in the reaction system according to the Bradford procedure (Bradford 1976). Fe(II) concentration was determined according to the method used by Dong et al. (2013). The dissolved oxygen was measured by dissolved oxygen analyzer (Rex JPBJ-608, Shanghai INESA, China). A pH meter (EL20, Shanghai Mettler-Toledo, China) was used to measure pH values. Gas samples were determined by a gas chromatography (GC7900, Techcomp, China) equipped with a thermal conductivity detector, a Porapak Q and a Molecular Sieve column. Helium was used as the carrier gas with a flow of 30 mL/min. The temperatures were 50 °C for the column, 120 °C for the detector and 120 °C for the injection port.

## Results

### Effects of His on the removal of nitrate and nitrite

**Fig. 1:**
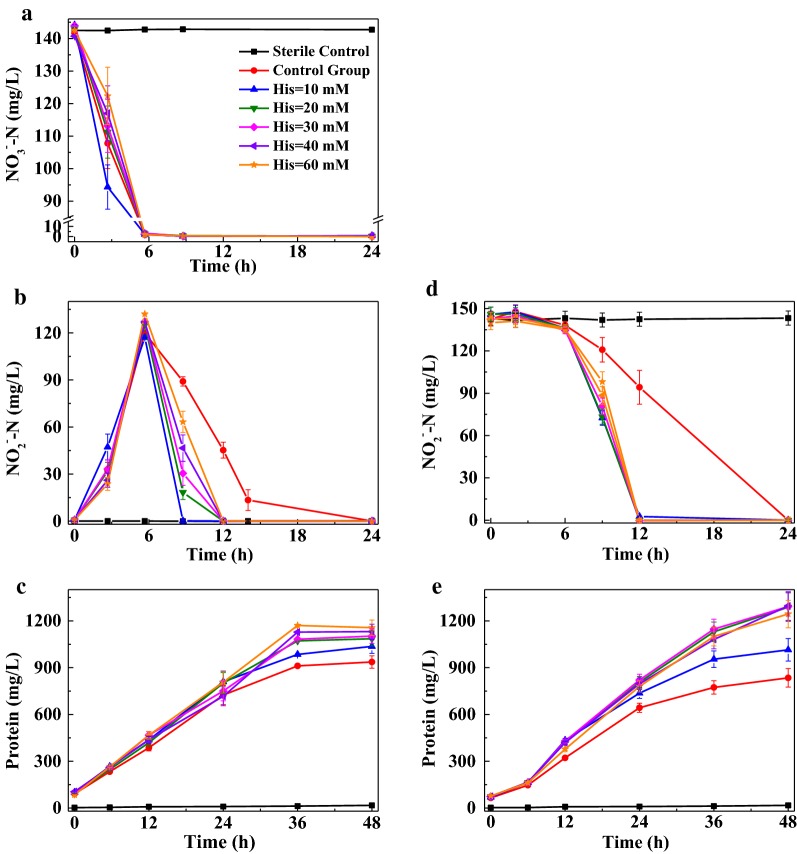
Effects of His on the removal of nitrate (**a**–**c**) and nitrite (**d**–**e**) under aerobic atmosphere by strain LYM

The effects of His on the removal of nitrate and nitrite by LYM were revealed in this part (Fig. 1). The reduction rate of nitrate and nitrite descended slightly with the rise of His concentration from 10 mM to 60 mM (Fig. 1a, d). Compared with the CG, 10 mM His facilitated the removal of nitrate and other concentrations showed adverse effects. The removal of nitrite accelerated under all His concentrations, regardless of whether the nitrite was added or generated (Fig. 1b, d). Besides, His slightly promoted the growth of LYM (Fig. 1c, e). As the toxicity of nitrite is higher than the toxicity of nitrate (Barreiros et al. 1998), the growth of LYM in the presence of nitrite (EG-nitrite) was delayed, compared to nitrate (EG-nitrate).

### Effects of initial His/Co^2+^ on the removal of nitrate and nitrite

**Fig. 2:**
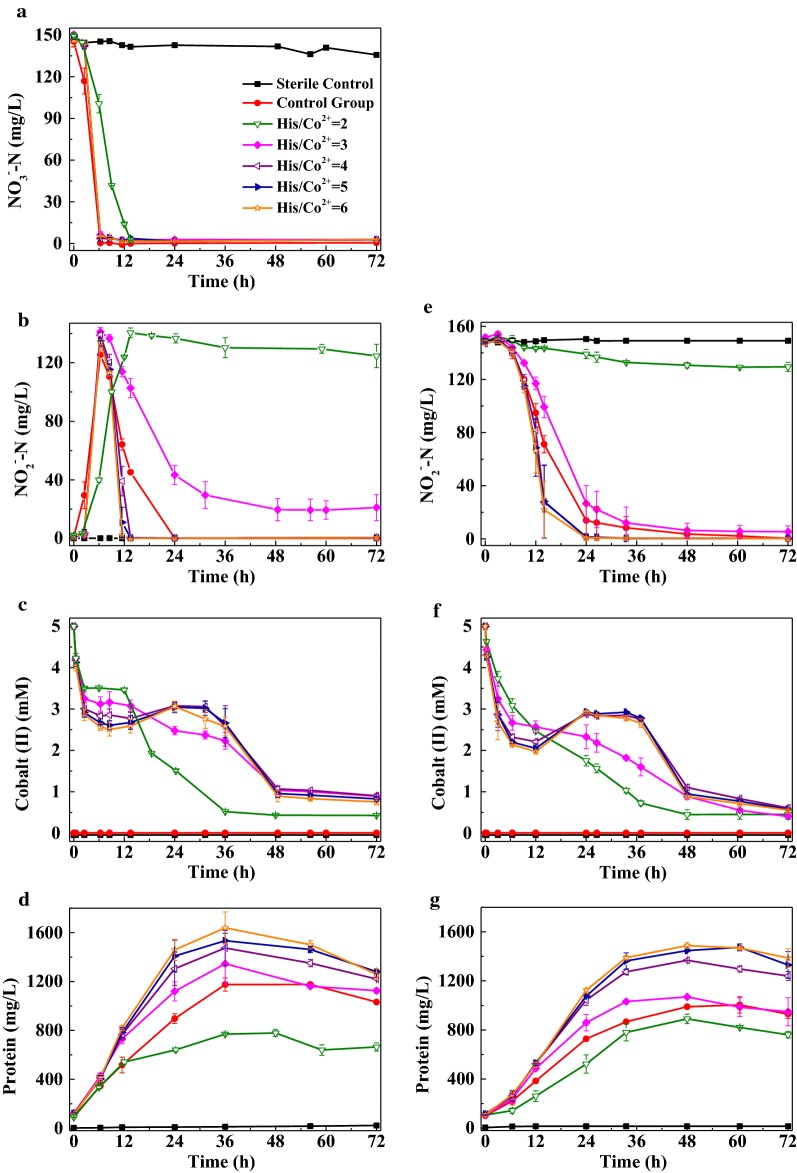
Effects of initial His/Co^2+^ on the removal of nitrate (**a**–**d**) and nitrite (**e**–**g**) under aerobic atmosphere by strain LYM

The initial His/Co^2+^ of CoHis absorbent presented significant effects on the removal of nitrate (Fig. 2a–d) and nitrite (Fig. 2e–g). Under the His/Co^2+^ of 2, 98% initial nitrate (149 mg L^−1^, 10.7 mM) was transferred to nitrite within 13.5 h (Fig. 2a), and only a small amount of the accumulated nitrite was further reduced (Fig. 2b). This experiment phenomenon, which was similar to that of nitrate aerobic denitrification by *Pseudomonas stutzeri* PCN-1 under 2.5 mg L^−1^ Cd(II), 5 mg L^−1^ Cu(II) or 10 mg L^−1^ Ni(II) (Gui et al. 2017), implied that the CoHis absorbent with a His/Co^2+^ of 2 had stronger inhibition effect on the activity of nitrite reductase than of nitrate reductase. In the EG-nitrite, the reduction of nitrite was very slow over the entire experiment, with a final removal efficiency of 13% (Fig. 2e). The concentrations of cobalt(II) dropped to 0.4 mM rapidly (Fig. 2c, f), with a plateau in 3–12 h in the EG-nitrate (Fig. 2c). The biomass of LYM was lower than that in the CG (Fig. 2d, g).

When the His/Co^2+^ rose to 3, LYM could transfer 96% initial nitrate (150 mg L^−1^, 10.7 mM) to nitrite within 6.3 h. As an intermediate of denitrification, nitrite accumulated to as much as 141 mg L^−1^ in 6.3 h, which was probably caused by the time lag between the highest activity level of nitrate reductase and nitrite reductase (Gui et al. 2017). The accumulated nitrite was reduced with a distinctly slower rate than in the CG, and the decrease of accumulated nitrite stopped at 20 mg L^−1^ after 48 h. The reduction of added nitrite was fast in 0–24 h with a rate slower than in the CG, then slowed down, and finally stopped at 6 mg L^−1^ after 48 h. The descent of cobalt(II) was fast in 0–6 h and slowed down in the following time, manifesting a plateau in the middle period. The biomass was higher than in the CG during the most of the process.

When His/Co^2+^ reached 4–6, 97% nitrate (147 mg L^−1^, 10.5 mM) was transferred to nitrite within 6.3 h. The accumulated and added nitrite was all reduced within 14 h (Fig. 2b) and 24 h (Fig. 2e), with a distinctly faster removal rate than in the CG. The cobalt(II) concentrations descended quickly in the first 3 h (Fig. 2c) and 6 h (Fig. 2f). In 3–31 h (His/Co^2+^ of 6 is 3–24 h in Fig. 2c) and 6–37 h, the descent of cobalt(II) apparently slowed down, meanwhile even slowly returned to around 2.9 mM. Then, the cobalt(II) concentrations continued to drop until 0.8 mM and 0.6 mM respectively. The biomass of LYM was well above the CG, and increased slightly with the rise of His/Co^2+^.

### Effects of CoHis absorbent on the removal of nitrate and nitrite

**Fig. 3:**
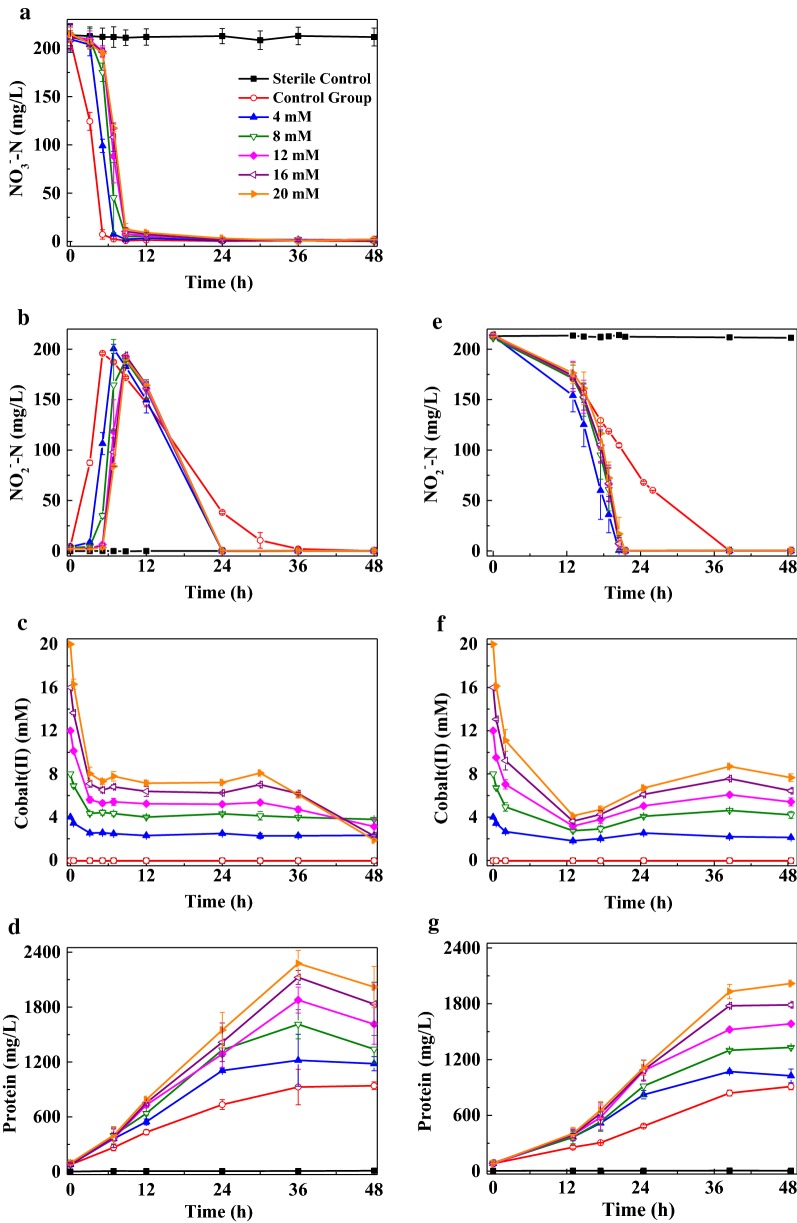
Effects of CoHis absorbent on the removal of nitrate (**a**–**d**) and nitrite (**e**–**g**) under aerobic atmosphere by strain LYM

This part was meant to study the influence of different concentrations of CoHis absorbent with an initial His/Co^2+^ of 4 on the removal of nitrate (Fig. 3a–d) and nitrite (Fig. 3e–g) by LYM under aerobic atmosphere, and the results would be used as reference for the future bioreactor experiments. Under CoHis absorbent, the peak removal rate of nitrate did not changed greatly compared with the CG, while the absorbent delayed the quick removal of nitrate. In 8.75 h, more than 94% nitrate was transferred to nitrite, which was further reduced within 24 h (Fig. 3a, b). CoHis absorbent with all concentrations could promote the removal of nitrite (Fig. 3e).

A quick drop of cobalt(II) concentration in EG-nitrate occurred at the beginning stage, and kept still for rather long time before the final descent (Fig. 3c). The variance of cobalt(II) concentration in EG-nitrite was similar, except that the end points of the first state became lower, and there were slight ascents after the quick drop (Fig. 3f). Additionally, in the oxidation experiment, the concentration of 5 and 10 mM cobalt(II) dropped continuously to 3.8 and 8.0 mM at 40 min, and to 1.6 and 3.0 mM at 280 min, respectively. Though Co(II)His was oxidized quickly (~ 70% was oxidized at 280 min), the oxidation rate was much lower than that of 5, 10 and 20 mM Fe(II)EDTA (above 97% was oxidized within 5 min). CoHis absorbent apparently facilitated the growth of LYM (Fig. 3d, g).

### Impacts of CoHis absorbent on gas products of aerobic denitrification

The above three experiments were conducted in open systems to investigate the effects on nitrate and nitrite reduction via quantifying nitrate, nitrite, cobalt(II) and biomass. In contrast, the experiment in this part was performed in closed system filled with oxygen to investigate the effects of CoHis absorbent on aerobic denitrification from the aspect of gas products by quantifying N_2_O and N_2_.

**Fig. 4:**
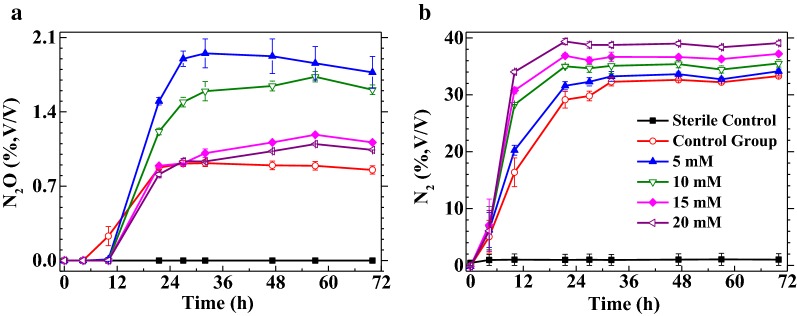
Effects of CoHis absorbent on gas products of nitrate reduction under aerobic atmosphere by strain LYM: **a** N_2_O, **b** N_2_

There was a small amount of N_2_O (0.9%, v/v) in the CG, similar with the report of Zhang et al. (2015). The accumulation of N_2_O was 1.8%, 1.6%, 1.1% and 1.0% (v/v) respectively under 5, 10, 15, and 20 mM CoHis absorbent (Fig. 4a). The produced N_2_ were 34.1%, 35.5%, 37.2% and 39.1% (v/v) under CoHis absorbent, a little bigger than 33.3% in the CG (Fig. 4b).

## Discussion

This study verified the applicability of Co(II)His in the CABR technology from the aspect of the biological reduction process. First, unlike the deleterious effects of free EDTA (Hau et al. 2008), His could promote the growth of LYM (Fig. 1c, e) as it is an amino acid constituting the material basis of life. His also benefited the aerobic denitrification process, especially the reduction of nitrite. The reason might be that His is a kind of important ligand in aerobic denitrification enzymes, participating in the coordination with the central atoms of enzymes (Brigé et al. 2002; Kumita et al. 2004; Kujime et al. 2008; Solomon et al. 2014; Maia and Moura 2014), and especially, it plays an important role in significantly promoting the catalytic performance of nitrite reductase (Kujime et al. 2008; Maia and Moura 2014).

As is known, the effect of a substance on microorganisms depends on its concentration. The relative response of microorganisms to the heavy metals of different concentrations is categorized into three zones: (a) zone of increasing stimulation (the stimulation effect increases with the rise of concentration), (b) zone of decreasing stimulation (the stimulation effect decreases with the rise of concentration) and (c) toxicity zone (the inhibition effect increases with the rise of concentration) (Gikas 2008). Though not experimentally researched, it still could be inferred that if the His concentration was well beyond the range of the present study, a similar response property of LYM to His would be seen. Accordingly, the effect of 10–60 mM His on nitrite reduction might well be located on the zone of decreasing stimulation. In contrast, its effects on nitrate reduction would be across the decreasing stimulation zone and the toxicity zone.

Secondly, the inhibition effect of CoHis absorbent on LYM could be substantially alleviated by adjusting the initial His/Co^2+^ to 4 or higher (Fig. 2). As mentioned above, the toxicity of heavy metals is strongly related to the free metal concentration (Hu et al. 2002; Semerci and Çeçen 2007). It is also believed that the first step in the microbial response to toxic heavy metals is the uptake of free metal cations, which are generally thought to be the most toxic metal species via a nonspecific metal inorganic transport system (Hu et al. 2002). Hence, the inhibition effect of CoHis absorbent with different initial His/Co^2+^ might stem from the residual Co^2+^ in the absorbent.

The reaction 5 used to prepare the CoHis absorbent is a reversible reaction with an equilibrium constant of 4 × 10^−7^ at 24 °C (Burk et al. 1946). The substance content in reaction 5 at different initial His/Co^2+^ when it reached equilibrium was obtained by theoretical calculation (Table 1). When the His/Co^2+^ rose from 2 to 4, the amount of the produced Co(II)His increased dramatically, thus leaving distinctly less Co^2+^ in the liquid. This variance essentially reduced or even eliminated the toxicity of the absorbent on bacteria. As the His/Co^2+^ continued rising, the residual Co^2+^ concentrations reduced slightly, resulting in similar removal characteristics of nitrate or nitrite to that at 4. Furthermore, affected by the excessive His in the CoHis absorbent, the nitrite reduction process was also promoted by apparent degree (Fig. 2b, e), which is similar to that presented in Fig. 1b, d. In general, based on the synthesized consideration of nitrogen removal efficiency, cobalt(II) reduction performance, and economic elements, the initial His/Co^2+^ for absorbent preparation was determined as 4.

**Table 1:** Theoretical calculation of the substance content in reaction 5 when it reached equilibrium at different initial His/Co^2+^ (T = 24 °C, pH = 7, initial cobalt(II) = 5 mM)

His/Co^2+^	Co(II)His/initial cobalt(II) (%)	Residual Co^2+^(mg/L)	Residual His (mM)
2	93.833	18.194	0.6
3	99.900	0.294	5
4	99.975	0.074	10
5	99.989	0.033	15
6	99.994	0.018	20

The equilibrium constant under 30 °C was not publicly available, thus the constant was referred from 24 °C

Then, at the initial His/Co^2+^ of 4, the influence of 0–20 mM CoHis absorbent on the removal of nitrate and nitrite by LYM under aerobic atmosphere was investigated, and LYM could tolerate 20 mM CoHis absorbent. By comparing the results in Figs. 1 and 3, it could be found that CoHis absorbent delayed the quick removal of nitrate (Fig. 3a) by a degree greater than what His EG (Fig. 1a) did. The average peak reduction rate of nitrite under 4–20 mM CoHis absorbent (Fig. 3e, 13–20 h: 21.6 ± 0.6 mg L^−1^ h^−1^) was approximate to that under 10–60 mM His (Fig. 1d, 6–12 h: 22.5 ± 0.2 mg L^−1^ h^−1^). The growth of LYM was facilitated much more remarkably by CoHis absorbent (Fig. 3d, g) than by His (Fig. 1c, e). Considering that the CoHis absorbent mainly contained CoHis and His (Table 2), it could be inferred that CoHis had slight inhibition effect on the reduction of nitrate, and this effect disappeared quickly once LYM adapted to CoHis environment. CoHis had no apparent effect on the reduction of nitrite, but facilitated the growth of LYM.

**Table 2:** Theoretical calculation of the substance content in reaction 5 when it reached equilibrium at different initial cobalt concentrations (T = 24 °C, pH = 7, His/Co^2+^ = 4)

Initial cobalt(II) (mM)	Co(II)His/initial cobalt(II) (%)	Residual Co^2+^(mg/L)	Residual His (mM)
4	99.961	0.092	8
5	99.975	0.074	10
8	99.990	0.046	16
10	99.994	0.037	20
12	99.996	0.031	24
15	99.997	0.025	30
16	99.998	0.023	32
20	99.998	0.018	40

The equilibrium constant under 30 °C was not publicly available, thus the constant was referred from 24 °C

It is inspiring to see that LYM could reduce Co(III)His in aerobic condition. In the oxidation experiment of Co(II)His, the concentration of cobalt(II) dropped continuously, due to the oxidation process of Co(II)His to Co(III)His by dissolved oxygen. While cobalt(II) concentration in the aerobic denitrification experiments showed different change trends (Figs. 2c, f, 3c, f), which might attribute to biological reduction process of Co(III)His to Co(II)His by LYM. The cobalt(II) concentrations in the aerobic denitrification experiments were dominated by both the oxidation process of Co(II)His to Co(III)His by the dissolved oxygen and the inverse biological reduction process of Co(III)His to Co(II)His by LYM. The capacity owned by LYM that could reduce nitrate, nitrite and Co(III)His simultaneously under aerobic conditions was beneficial for the regeneration of the absorbent. Reddy et al. (2016) also realize the simultaneous reduction of nitrate and Co(III)EDTA by denitrifying granular sludge.

Last but not least, also at the initial His/Co^2+^ of 4, the influence of 0–20 mM CoHis absorbent on gas products of aerobic denitrification was investigated. In view that N_2_ was the major product (95.0%, 95.7%, 97.1% and 97.5% in the sum of N_2_O and N_2_), it could be affirmed that, as high as 20 mM, CoHis absorbent could not lead to the accumulation of N_2_O by massive amount. The result that the accumulation of N_2_O was a bit higher than in the CG illustrated that CoHis absorbent showed slight inhibition on the reduction of N_2_O. Moreover, the inhibition effect became weaker with the increase of CoHis absorbent concentration. In view of that, only the concentration of Co^2+^ decreased with the rise of CoHis absorbent concentration (Table 2), the reduction of N_2_O probably was inhibited by the small amount of residual Co^2+^. At the same time, the reduction of N_2_O might probably be promoted by residual His in the absorbent for His participates in the coordination with the central atom of nitrous oxide reductase (Solomon et al. 2014). As N_2_O is a potent greenhouse gas, refining the experimental conditions and thus eliminating the accumulation of N_2_O would still be an important point in the future research.

It is reported that cobalt has been identified as micronutrient at trace concentrations; however, it is microbial growth inhibitor at relatively high concentrations (Gikas 2008). In this study, when cobalt was in presence as CoHis and as high as 20 mM, it still showed to be beneficial for the growth of LYM and harmless to bioactivity. This result proves once again that the toxicity of cobalt on microorganism can be reduced by coordination with ligands (Bartacek et al. 2008), and provided a positive evidence for the application of CoHis in the CABR process. For the future CABR bioreactor experiments, the concentration of CoHis absorbent could be set as 20 mM, which was basically equivalent to the concentrations of ferrous complexes in the CABR process (Zhang et al. 2008; Liu et al. 2012).

In summary, His could significantly promote the removal of nitrite by *P. versutus* LYM. The inhibition effect of CoHis absorbent on LYM could be substantially alleviated by adjusting the initial His/Co^2+^ to 4 or higher. At His/Co^2+^ of 4, 4–20 mM CoHis presented no distinct effect on nitrite reduction, but slightly inhibited the reduction of nitrate, and obviously promoted the growth of LYM. The main gas product of aerobic denitrification was N_2_ under 5–20 mM CoHis absorbent. The concentration of CoHis in the absorbent could be determined as 20 mM with an initial His/Co^2+^ of 4 in the future CABR bioreactor experiments with CoHis as absorbent.

## Data Availability

The data supporting the conclusions of this article is included within the article. Data and materials can also be requested from the corresponding author.
